# Factors Associated with Utilization of Dipeptidyl-4 Inhibitors in Patients with Type 2 Diabetes Mellitus: A Cross-Sectional Retrospective Study

**DOI:** 10.1155/2014/367564

**Published:** 2014-09-02

**Authors:** Hasniza Zaman Huri, NorFarahen Selamat, Shireene Ratna Vethakkan

**Affiliations:** ^1^Department of Pharmacy, Faculty of Medicine, University of Malaya, 50603 Kuala Lumpur, Malaysia; ^2^Clinical Investigation Centre, Faculty of Medicine, University Malaya Medical Centre, 13th Floor Main Tower, 59100 Lembah Pantai, Kuala Lumpur, Malaysia; ^3^Endocrinology Unit, Department of Medicine, Faculty of Medicine, University of Malaya, 50603 Kuala Lumpur, Malaysia

## Abstract

Dipeptidyl-4 (DPP-4) inhibitors are oral antidiabetic agents recently introduced to Malaysia. Thus, limited data is available on their utilization patterns and factors associated with their use. This study aims to analyse the utilization patterns of DPP-4 inhibitors, factors that influenced the choice of agent, and the rationale for treatment with DPP-4 inhibitors in patients with type 2 diabetes mellitus. This retrospective study was conducted to address the utilization pattern of DPP-4 inhibitors and factors that influence choice in type 2 diabetes mellitus patients. 299 subjects taking either sitagliptin or vildagliptin from September 2008 to September 2012 were included in the study. Sitagliptin was more frequently prescribed than vildagliptin. Of the patients prescribed DPP-4 inhibitors, 95% received combinations of these and other agents, whereas only 5% were prescribed DPP-4 inhibitors as monotherapy. Factors affecting the utilization of DPP-4 inhibitors included age (*P* = 0.049) and concomitant use of beta blockers (*P* = 0.045) and aspirin (*P* = 0.008). Early identification of factors associated with DPP-4 inhibitors is essential to enhance quality use of the drugs.

## 1. Introduction

The International Diabetes Federation (IDF) has estimated that 371 million people worldwide had diabetes in 2012 and that 552 million will have this disease by 2030 [1]. Moreover, the costs involved in managing patients with diabetes worldwide were estimated to be approximately 471 billion USD. At least 90% of individuals with diabetes have type 2 diabetes mellitus (T2DM) [2]. In Malaysia, the prevalence of T2DM has increased markedly, from 11.6% of the population in 2006 to 15.2% in 2011, a relative increase of 31% over 5 years [3]. A 2007 report stated that antidiabetic medications were the second highest healthcare expenditures for medications in Malaysia [4].

According to the American Diabetes Association (ADA), management of T2DM involves a combination of life-style modifications and pharmacological approaches, consisting of oral antidiabetic (OAD) agents and insulin injection [5]. At present, six classes of OADs are used to treat patients with T2DM, with dipeptidyl peptidase-4 (DPP-4) inhibitors being one of the newer drug classes. DPP-4 inhibitors inhibit the degradation of the hormone GLP-1, which stimulates insulin release immediately after a meal. Currently available DPP-4 inhibitors include sitagliptin, vildagliptin, saxagliptin, alogliptin, and linagliptin, which differ in pharmacokinetic and pharmacodynamics profiles [6]. Generally, OADs have excellent safety profiles, with a low rate of adverse effects [7].

Sitagliptin was the first DPP-4 inhibitor approved for use in Malaysia to treat patients with T2DM [8]. At present, three DPP4 inhibitors are available for use in Malaysia: sitagliptin, saxagliptin, and vildagliptin [9], but their use is not widespread. These agents are more likely to be prescribed to patients in private health institutions than in government hospitals and clinics [4]. Guidelines for the appropriate utilization of these agents include those of the National Institute for Health and Clinical Excellence (NICE) and the Scottish Medicine Consortium (SMC). However, there are no specific guidelines in Malaysia on the appropriate use of these drugs. Thus, it is important to determine factors that may influence physicians to prescribe DPP-4 inhibitors.

Little is known about the utilization pattern of DPP-4 inhibitors, both within Malaysia and in other countries. This study was, therefore, designed to determine the patterns of DPP-4 inhibitor utilization and factors that influence their utilization.

## 2. Materials and Methods

### 2.1. Sample Population

The study population consisted of all UMMC patients aged ≥18 years who were diagnosed with T2DM and had received sitagliptin or vildagliptin at any time from September 2008 to September 2012.

### 2.2. Study Procedures

This retrospective, cross-sectional study was performed in accordance with the Declaration of Helsinki and was approved by the medical ethics committee (MEC) of UMMC (reference number 956.29), which waived the requirement for written informed consent from the participants. Patients were included if they were aged ≥18 years, fulfilled the requirements of the ICD-10 code for T2DM (E11.0–E11.9), and were prescribed either sitagliptin or vildagliptin at any time from September 2008 to September 2012, either as monotherapy or in combination with other antidiabetic medications. Patients were excluded if they had been diagnosed with type 1 diabetes mellitus (T1DM), had never received any DPP4 inhibitor, or were diagnosed with a psychiatric illness that may compromise compliance with diabetic treatment.

Demographic and clinical parameters were collected for each eligible patient. Demographic parameters included age, gender, ethnicity, weight, height, and body mass index (BMI). Characteristics of T2DM included the year of diagnosis, HbA1c concentration, and either FPG or RPG concentration. Characteristics of T2DM treatment included class and name of drug and dosage and date of prescribed DPP-4 inhibitors.

Other clinical parameters included the number and type of comorbidities, with comorbidities defined as chronic diseases requiring long-term treatment; concurrent medications, including medications prescribed to manage comorbidities; renal impairment, defined as creatinine clearance <50 mL/min [10], and hepatic impairment, defined as chronic hepatitis, liver cirrhosis, or elevation of liver enzymes such as alanine transaminase (ALT) and aspartate transaminase (AST) to more than 3 times the upper limit of normal value.

### 2.3. Statistical Methods

#### 2.3.1. Determination of Sample Size

The sample size required was calculated using Epi Info Program Version 7.0 (CDC, Atlanta, GA, USA). The level of significance, *α*, was set as 0.05 and the desired power of the study (1 − *β*) was 80%. Assuming an expected proportion of T2DM patients of 20.8% and a confidence limit of 5%, the minimum number of patients required was 108.

All data extracted were analyzed using the Statistical Package for Social Sciences (SPSS) software version 20.0 (Armonk, New York, USA). All continuous data were tested for normality using the Kolmogorov-Smirnov test. Normally distributed parameters were expressed as mean ± standard deviation, and nonnormally distributed parameters were expressed as median and range. Categorical data were analyzed by Pearson's chi square test or Fisher's exact tests, as appropriate. A *P* value < 0.05 was considered statistically significant.

## 3. Results

### 3.1. Study Subject's Disposition

Convenience sampling identified 370 eligible subjects, but the medical records for only 316 of these subjects (85.4%) could be successfully retrieved from the Medical Records Unit (MRU) of UMMC. Of these 316 patients, 17 were excluded for serious psychiatric illness. Thus, 299 patients were analyzed.

### 3.2. Demographic Characteristics

Of the 299 subjects, 133 (44.5%) were males and 166 (55.5%) were females. Ethnically, 103 subjects (34.4%) were Chinese, 99 (33.1%) were Malay, and 97 (32.4%) were Indian. Mean subject age was 63.1 ± 11.4 years (range, 22 to 89 years), with the Kolmogorov-Smirnov test showing a normal distribution by age. We found that 161 subjects (53.8%) were <65 years old and 138 (46.2%) were aged ≥65 years.

Height and weight were available for only 220 subjects (73.6%). BMI was not normally distributed in these 220 subjects, with median BMI of 26.9 kg/m^2^ (range, 18.4 kg/m^2^ to 49.3 kg/m^2^). BMI was classified into four categories: underweight (<18.5 kg/m^2^), normal weight (18.5–22.9 kg/m^2^), overweight (23–27.4 kg/m^2^), and obese (≥27.5 kg/m^2^). Using these criteria, 99 subjects (33.1%) were overweight and 90 (30.1%) were obese (Table 1).

### 3.3. Clinical Characteristics

Information on duration of T2DM since diagnosis was available for only 220 (73.6%) patients. The Kolmogorov-Smirnov test showed that the duration of T2DM for these subjects was not normally distributed. The median duration was 13.5 years (range, 2 to 37 years). In most subjects (77.6%), the duration of T2DM was >10 years.

Information on HbA1c concentration was available for 266 (89%) of the 299 patients. HbA1c concentration in these 266 patients was not normally distributed. Median HbA1c concentration was 7.75% (range, 5.2% to 19.4%), with more than 89% of the subjects having HbA1c concentrations ≥6.5% (Table 2).

Of the 299 subjects, 255 (85.3%) were taking 5 or more concurrent medications. Hypertension and hyperlipidemia were the most frequent comorbidities, observed in 251 (83.9%) and 210 (70.2%) subjects, respectively, followed by obesity, observed in 86 subjects (28.8%). Not surprisingly, the most frequently prescribed concurrent medications were HMG-CoA inhibitors and salicylates, prescribed to 251 (83.9%) and 131 (43.8%) subjects, respectively. Diuretics included thiazides (27.8%), furosemide (11.0%), and spironolactone (2.3%).

### 3.4. Pattern of Use of DPP-4 Inhibitors

Sitagliptin was the DPP-4 inhibitor most frequently utilized at UMMC, prescribed to 86% of patients, with vildagliptin prescribed to the other 14%. The most frequent doses of sitagliptin were 100 mg OD (58.7%) and 50 mg OD (28.4%). The combination of sitagliptin/metformin in one tablet was prescribed to 50% of patients, with 28.6% taking a dosage regimen of 50/850 mg OD and 21.4% taking 50/500 mg OD. Vildagliptin was available only as 50 mg tablets, with most patients taking this dose OD. The two fixed dose combinations of vildagliptin/metformin, both given twice daily, were equally preferred (36.8%).

Both DPP-4 inhibitors were administered in combination with other antidiabetic medications. Only 4.3% of patients were prescribed sitagliptin monotherapy and only 2.4% were prescribed vildagliptin monotherapy. Combinations of DPP-4 inhibitors with metformin and sulfonylureas were prescribed to 224 (74.9%) and 209 (69.9%) of the subjects, respectively. Fewer DPP-4 treated patients were also treated with thiazolidinediones (TZD), meglitinides, insulin, and acarbose.

### 3.5. Factors Significantly Associated with Utilization of DPP-4 Inhibitors

#### 3.5.1. Age

The majority of patients taking either sitagliptin or vildagliptin were aged <65 years. A significant association between age and DPP-4 inhibitor use was observed (*P* = 0.049) (Table 3).

#### 3.5.2. Concurrent Medications

*(1) Beta Blockers.* Of the 299 subjects, 93 (31.1%) were taking a beta blocker along with a DPP-4 inhibitor. A significant association was observed between utilization of DPP-4 inhibitors and beta blockers (*P* = 0.045) (Table 4).

*(2) Aspirin.* Of the 299 patients, 131 (43.8%) were taking aspirin concomitantly. Aspirin use was significantly associated with prescription of DPP-4 inhibitors (Table 5).  Table 6 shows parameters not significantly associated with the use of DPP-4 inhibitors.

## 4. Discussion

### 4.1. Clinical Characteristics

We found that the time from diagnosis of T2DM to treatment with DPP-4 inhibitors was longer than 10 years in 69% of the subjects, with a median duration of 13.5 years, longer than the mean 10.8 years and 9.27 years reported in earlier studies [11, 12]. However, the duration of T2DM could not be determined in 79 of the 299 (26.4%) subjects, which may have altered the median duration in the entire cohort.

Glycemic control, as indicated by HbA1c level, was poorer in patients treated at UMMC than in other studies. We found that 13% of subjects assessed for HbA1c had HbA1c levels <6.5%, similar to findings showing that 11.4% and 11.6% of patients with T2DM had HbA1c levels <6.5% [13, 14]. In contrast, a cross-sectional survey reported blood glucose control (HbA1c < 6.5%) in 18% of patients, but HbA1c concentrations were measured in only 52.6% of that patient cohort [15].

Hypertension was the most frequent comorbidity in our patient population, followed by hyperlipidemia, obesity, and heart problems. A previous study reported similar results for hypertension and hyperlipidemia, but since overweight and obesity were pooled, a comparison with our results was impossible [15]. In addition, Mafauzy reported that hypertension was the most common comorbid condition in diabetic patients [16]. The Malaysian NHMS IV conducted in 2011 reported that hyperlipidemia (35.1%) and hypertension (32.7%) were the most prevalent noncommunicable diseases [17]. However, the NHMS was a survey of the entire population, not only of patients with T2DM.

We found that more than 85% of the subjects in this study were taking five or more medications (polypharmacy) and that older age was significantly associated with polypharmacy (*P* = 0.000). HMG-CoA inhibitors (statins) were the most frequently drug class prescribed concomitantly to these subjects. More than 97% of our subjects were aged ≥40 years and were given statins regardless of baseline LDL concentration [18]. Moreover, a local study performed at a university primary care center reported that statins (69%) were the class of drugs most frequently prescribed concomitantly to diabetic patients [16]. The same study reported that 33% of patients were prescribed salicylate, likely because of the relatively low percentage of patients with coronary artery disease (9.9%) and stroke (5.2%). A cross-sectional study found that cardiovascular drugs were the most frequently prescribed to 27.3% of patients [19].

We found that the four leading classes of antidiabetic agents prescribed to our T2DM patients were metformin, sulphonylureas, insulin, and acarbose, which were given to 224 (74.9%), 209 (69.9%), 70 (23.4%), and 41 (13.7%) subjects, respectively. This finding is in good agreement with a previous study showing that the four classes of drugs most prescribed for patients with T2DM were metformin (84%), sulphonylureas (81%), insulin (16%), and acarbose (8%) [16].

### 4.2. Utilization Pattern of DPP-4 Inhibitors by T2DM Patients

Two DPP-4 inhibitors were used to treat T2DM patients at UMMC, sitagliptin and vildagliptin. More than 85% of our patients were prescribed sitagliptin, whereas only 15% received vildagliptin, similar to findings showing that 57 of 66 subjects (86.4%) prescribed a DPP-4 inhibitor were taking sitagliptin [20]. At the time of this study, these two agents were the only DPP-4 inhibitors available at UMMC, although, currently, saxagliptin and linagliptin are also available in Malaysia. Sitagliptin was the first DPP-4 inhibitor approved for use in Malaysia in June 2007 [9]. Although vildagliptin has been licensed for use in Malaysia, it has not yet been approved by the U.S. FDA. Sitagliptin and vildagliptin are comparable clinically, both in effectiveness and incidence of hypoglycemia [21]. Sitagliptin is available as a single drug, at doses of 25 mg, 50 mg, and 100 mg, and in combination with metformin, at a dose of 50 mg. Vildagliptin is available at one dosage (50 mg), both as a single drug and in combination with metformin. Assessment of dosage regimens of sitagliptin monotherapy found that 100 mg once daily was the preferred dosage, administered to 58.7% of patients, in line with dosage recommended by the Malaysian Clinical Practice Guideline on Management of Type 2 Diabetes Mellitus [22]. In contrast, 50% of subjects taking sitagliptin/metformin were not prescribed the dosage recommended in the guidelines.

The Malaysian Drug Formulary for 2013 recommends that the dose of vildagliptin be 50 mg BD if taken with metformin and 50 mg OD if taken with sulphonylureas [9]. When combined with metformin, the Formulary recommends a maximum daily dose of 100 mg vildagliptin and 2000 mg metformin. Most of the subjects in this study prescribed vildagliptin were given appropriate dosage regimens.

Few studies to date have assessed patterns of utilization of DPP-4 inhibitors or of individual agents. Thus, our results can only be compared with general utilization patterns of OAD agents. A study of patients hospitalized at a tertiary care referral hospital found that the most widely used OAD was biguanides (23%), followed by sulphonylureas (22.5%) and thiazolidinediones (11%) [23]. Only 9.5% of those patients were treated with DPP-4 inhibitors.

Combinations of DPP-4 inhibitors with metformin and sulphonylureas were the most popular, followed by combinations with insulin. Sitagliptin can be prescribed as monotherapy as well as in combination with metformin, a sulphonylurea, or a thiazolidinedione [9, 22], although taking these combinations may enhance the risk of hypoglycemia. Guidelines have recommended that vildagliptin be used as second-line therapy in combination with either metformin or sulphonylureas whenever the latter two agents are not sufficient to provide glycemic control or are not tolerated [9, 24].

### 4.3. Factors Significantly Associated with the Use of DPP-4 Inhibitor in Type 2 Diabetes Mellitus

#### 4.3.1. Age

We found that age was weakly associated with the utilization of DPP-4 inhibitors with patients aged ≥65 years more likely to be given DPP-4 inhibitors (*P* = 0.049). The Malaysian Drug Formulary 2013 has recommended the use of sitagliptin in T2DM patients, especially in elderly patients with multiple comorbidities who frequently experience hypoglycemia while on other OADs. Vildagliptin has been recommended for T2DM patients with poor glycemic control on the maximal tolerated dose of metformin monotherapy and at high risk of hypoglycemia [9]. Sitagliptin and vildagliptin were well tolerated by elderly patients and are associated with a low risk of hypoglycemia, as well as having similar efficacy in both older and younger patients [25]. However, the association between these variables was very weak, which may be due to the limited use of DPP-4 inhibitors in elderly individuals. These drugs are rather costly, limiting their availability to older individuals. Indeed, we found that the majority of patients in our study (53.8%) were nonelderly.

#### 4.3.2. Concurrent Medications

This study found significant associations between the use of DPP-4 inhibitors and the concomitant use of beta blockers (*P* = 0.045). Most subjects taking sitagliptin and vildagliptin were not prescribed beta blockers, in line with recommendations that beta blockers are prescribed to T2DM patients with hypertension only when alternative agents cannot be used or when there are concomitant compelling indications (e.g., effort angina, tachyarrhythmias, and previous myocardial infarction) [18]. The effects of beta blockers in patients with T2DM are nonselective and may include masking the early symptoms of hypoglycemia and slowing recovery from hypoglycemia attacks [18]. Nevertheless, three beta blockers, bisoprolol, carvedilol, and metoprolol succinate, have been recommended for T2DM patients with heart failure. Indeed, an observational study in patients with T2DM and systolic heart failure found that carvedilol and bisoprolol significantly improved glycemic control [26].

The concurrent use of aspirin was found to be negatively associated with the use of DPP-4 inhibitors (*P* = 0.008), in that less aspirin was prescribed to patients taking either sitagliptin or vildagliptin. This may be due to the relatively low prevalence of comorbidities that warrant the use of aspirin as treatment or prophylaxis. The three most common comorbidities in our patient cohort were hypertension, hyperlipidemia, and obesity, all three of which do not require treatment or prophylaxis with aspirin [18, 27, 28]. Nevertheless, aspirin was the second most frequent drug prescribed to our subjects, being taken by more than 43%. This was likely due to the high proportion of our patients with ischemic heart disease (IHD) and cerebrovascular accidents (CVA).

### 4.4. Factors Not Significantly Associated with Utilization of DPP-4 Inhibitors

#### 4.4.1. Duration of Diabetes

As for duration since the diagnosis of T2DM, both sitagliptin and vildagliptin had a higher proportion of subjects that had the duration since diagnosis of T2DM of more than 10 years. Despite this, the association failed to achieve significance level of *P* < 0.05 (*P* = 0.661). Besides, there was no literature found that studies the effect of these factors on prescribing of DPP-4 inhibitors. There was also no guideline found that advocates the use of DPP-4 inhibitors according to the duration of T2DM diagnosis. Therefore, it reflects that duration since the diagnosis of T2DM did not influence the prescriber in prescribing DPP-4 inhibitors.

#### 4.4.2. HbA1C

World Health Organization has announced in 2011 that the new level of HbA1c <6.5% is the new cut-off point for diagnosing diabetes [29]. Formerly, it was agreed that the level of HbA1c was less than 7% as a diagnostic criterion. HbA1c level is used as an indicator that reflects the glycemic control for the past 2-3 months. An individual with A1c of less than 6.5% is considered to have achieved good glycemic control. In this study, the same parameter with the newly recommended cut-off point was used. Subjects on both sitagliptin and vildagliptin had a poor glycemic control with A1c level equal to or more than 6.5%. However, when tested for association, this parameter failed to be associated significantly with the use of DPP-4 inhibitors (*P* = 0.995). Hence, glycemic control, as demonstrated by A1c level, was not a determinant in prescribing DPP-4 inhibitors.

#### 4.4.3. BMI

As for the BMI, most of the overweight and obese patients were taking sitagliptin and vildagliptin. There were nonsignificant associations between utilisation of sitagliptin and BMI. According to the Malaysian guidelines, the first-line treatment option for obese type 2 diabetes patients is metformin [22]. As other oral antidiabetic agents are acceptable alternatives to metformin, other OADs inclusive DPPIV inhibitors will be used in a case of metformin intolerability/ineffectiveness and contraindicated in overweight and obese patients. Therefore, DPPIV inhibitors would not be selected by the prescribers as the first-line treatment in overweight and obese patients. Metformin will still be the mainstay treatment for these patients.

## 5. Conclusions

Factors identified as significantly associated with prescribing DPP-4 inhibitors in this study included patient age and concurrent use of beta blockers and aspirin. Identification of factors underlying the use of DPP-4 inhibitors may enhance rational use of drugs and, thus, diabetes care in T2DM patients.

## 6. Limitation of Study

This study had several limitations, including its retrospective design. Only the subject characteristics affecting the prescribing of DPP-4 inhibitors were analyzed. In contrast, physician-associated factors were not assessed. In addition, since all information came from patient's medical records, errors may be caused by the absence of information or incorrect information.

## Figures and Tables

**Table 1:** Demographic characteristics of the patients.

Demographic characteristic	*N*	Number of patients (percentage, %)
Gender		
Male	299	133 (44.5)
Female	299	166 (55.5)
Age		
Nonelderly	299	161 (53.8)
Elderly	299	138 (46.2)
Ethnicity		
Malay	299	99 (33.2)
Chinese	299	103 (34.4)
Indian	299	97 (32.4)
BMI		
Underweight	220	1 (0.4)
Normal body weight	220	30 (13.6)
Overweight	220	99 (45.0)
Obese	220	90 (41.0)

**Table 2:** Clinical characteristics of the patients.

Clinical characteristics	*N*	Number of patients (percentage, %)
Duration since the diagnosis of T2DM		
≤10 years	220	67 (22.4)
11–20 years	220	98 (32.8)
21–30 years	220	46 (15.4)
>30 years	220	9 (3.0)
HbA1c		
Less than 6.5%	266	29 (10.9)
6.5% or more	266	237 (89.1)
Number of concurrent medication		
No polypharmacy	299	44 (14.7)
Polypharmacy	299	255 (85.3)

**Table 3:** Association between age and use of DPP-4 inhibitors.

Agent	*n*	Age	
		Nonelderly	Elderly
Sitagliptin	257	132 (51.4%)	125 (48.6%)
Vildagliptin	42	29 (69.0%)	13 (31.0%)

Chi square = 3.860, df = 1, *P* = 0.049.

**Table 4:** Association between use of a DPP-4 inhibitor and a beta blocker.

Agent	*n*	Beta blocker	
		Yes	No
Sitagliptin	257	86 (33.5%)	171 (66.5%)
Vildagliptin	42	7 (16.6%)	35 (83.4%)

Chi square = 4.001, df = 1, *P* = 0.045.

**Table 5:** Association between use of a DPP-4 inhibitor and aspirin.

Agent	*n*	Aspirin	
		Yes	No
Sitagliptin	257	121 (47.1%)	136 (52.9%)
Vildagliptin	42	10 (23.8%)	32 (76.2%)

Chi square = 7.025, df = 1, *P* = 0.008.

**Table 6:** Parameters not significantly associated with the use of DPP-4 inhibitors.

Patient characteristic	Number of patients (percentage, %)		*P* value
	Sitagliptin (*n* = 257)	Vildagliptin (*n* = 45)	
Gender			
Male	111 (43.2%)	22 (52.4%)	0.345^a^
Female	146 (56.8%)	20 (47.6%)	
Ethnicity			
Malay	85 (33.1%)	14 (33.3%)	0.345^a^
Chinese	85 (33.1%)	18 (42.9%)	
Indian	87 (33.9%)	10 (23.8%)	
BMI			
Underweight	0 (0%)	1 (4.0%)	0.160^a^
Normal weight	28 (14.4%)	2 (8.0%)	
Overweight	87 (44.6%)	12 (48.0%)	
Obese	80 (41.0%)	10 (40.0%)	
Duration since the diagnosis of T2DM			
≤10 years	58 (29.6%)	9 (37.5%)	0.661^a^
11–20 years	88 (44.9%)	10 (41.7%)	
21–30 years	41 (20.9%)	5 (20.8%)	
>30 years	9 (4.6%)	0 (0.0%)	
A1c			
<6.5%	25 (10.7%)	4 (12.5%)	0.762^b^
≥6.5%	209 (89.3%)	28 (87.5%)	
Polypharmacy			
<5 drugs	38 (14.8%)	6 (13.3%)	1.000^a^
≥5 drugs	219 (85.2%)	36 (86.7%)	
Renal impairment	70 (27.2%)	8 (19.0%)	0.352^a^
Hepatic impairment	6 (2.3%)	0 (0%)	1.000^b^
Heart disease	77 (29.9%)	9 (21.4%)	0.343^a^
Obesity	77 (29.9%)	9 (21.4%)	0.343^a^
Hypertension	219 (85.2%)	32 (76.19%)	0.211^a^
Hyperlipidemia	180 (70.0%)	30 (71.4%)	1.000^a^
Metformin	188 (73.1%)	36 (85.7%)	0.121^a^
Sulphonylurea	179 (69.6%)	30 (71.4%)	0.959^a^
Acarbose	34 (13.2%)	7 (16.6%)	0.720^a^
Thiazolidinedione	1 (0.39%)	0 (0%)	1.000^b^
Meglitinides	1 (0.39%)	1 (2.3%)	0.262^b^
Insulin	65 (25.3%)	5 (11.9%)	0.089^a^
ACE inhibitor	88 (34.2%)	19 (45.2%)	0.228^a^
ARB	95 (36.9%)	11 (26.1%)	0.238^a^
CCB	111 (43.2%)	19 (45.2%)	0.936^a^
Thiazide	74 (28.8%)	9 (21.4%)	0.422^a^
Loop diuretics	31 (12.0%)	2 (4.7%)	0.194^b^
Spironolactone	7 (2.7%)	0 (0%)	0.599^b^
Statin	215 (83.6%)	36 (85.7%)	0.912^a^
Fibrates	27 (10.5%)	4 (9.5%)	1.000^b^

^a^By Pearson chi square.

^b^By Fisher's exact test.
